# Substrate Utilization and Competitive Interactions Among Soil Bacteria Vary With Life-History Strategies

**DOI:** 10.3389/fmicb.2022.914472

**Published:** 2022-06-09

**Authors:** Ying Wang, Roland C. Wilhelm, Tami L. Swenson, Anita Silver, Peter F. Andeer, Amber Golini, Suzanne M. Kosina, Benjamin P. Bowen, Daniel H. Buckley, Trent R. Northen

**Affiliations:** ^1^Environmental Genomics and Systems Biology Division, Lawrence Berkeley National Laboratory, Berkeley, CA, United States; ^2^School of Integrative Plant Science, Cornell University, Ithaca, NY, United States; ^3^Joint Genome Institute, Lawrence Berkeley National Laboratory, Berkeley, CA, United States; ^4^Department of Microbiology, Cornell University, Ithaca, NY, United States

**Keywords:** genomics, life-history strategy, exometabolomics, resource competition, cross-feeding, *rrn* copy number

## Abstract

Microorganisms have evolved various life-history strategies to survive fluctuating resource conditions in soils. However, it remains elusive how the life-history strategies of microorganisms influence their processing of organic carbon, which may affect microbial interactions and carbon cycling in soils. Here, we characterized the genomic traits, exometabolite profiles, and interactions of soil bacteria representing copiotrophic and oligotrophic strategists. Isolates were selected based on differences in ribosomal RNA operon (*rrn*) copy number, as a proxy for life-history strategies, with pairs of “high” and “low” *rrn* copy number isolates represented within the Micrococcales, Corynebacteriales, and Bacillales. We found that high *rrn* isolates consumed a greater diversity and amount of substrates than low *rrn* isolates in a defined growth medium containing common soil metabolites. We estimated overlap in substrate utilization profiles to predict the potential for resource competition and found that high *rrn* isolates tended to have a greater potential for competitive interactions. The predicted interactions positively correlated with the measured interactions that were dominated by negative interactions as determined through sequential growth experiments. This suggests that resource competition was a major force governing interactions among isolates, while cross-feeding of metabolic secretion likely contributed to the relatively rare positive interactions observed. By connecting bacterial life-history strategies, genomic features, and metabolism, our study advances the understanding of the links between bacterial community composition and the transformation of carbon in soils.

## Introduction

The transformation of soil organic carbon (C) by microorganisms plays a critical role in determining the long-term fate of soil C (Schimel and Schaeffer, 2012; Liang et al., 2017). To improve soil C projections, global C cycle models have increasingly incorporated knowledge of microbial life-history traits (Wieder et al., 2015). Trait-based theories can be used to classify microorganisms into ecologically relevant functional groups, such as the zymogenous–autochthonous and *r*- vs. *K*-selection strategists (Prosser et al., 2007). Analogous to these concepts is the widely applied copiotroph–oligotroph classification framework, where copiotrophs are thought to be adapted to resource-rich conditions with higher nutrient demand and faster growth rates while oligotrophs are thought to be better adapted to resource-poor conditions and exhibit traits of slow but efficient growth (Fierer et al., 2007; Roller and Schmidt, 2015). The growth rate vs. efficiency trade-off between copiotrophic and oligotrophic bacteria has been shown to associate with several genomic features in bacteria (Lauro et al., 2009; Roller et al., 2016). Specifically, ribosomal RNA operon (*rrn*) copy number is positively correlated with growth rate which is negatively correlated with efficiency, indicating the potential of using genomic signatures to identify the life-history traits of bacteria (Roller et al., 2016).

A limited number of studies have characterized how soil microorganisms from different ecological groups differ in their C metabolism, including consumption and production of diverse simple organic molecules. The ability of microorganisms to metabolize specific organic substrates can constrain their growth rate and efficiency (Goldfarb et al., 2011; Saifuddin et al., 2019; Muscarella et al., 2020). However, it remains not well-understood how substrate use differs across the copiotroph–oligotroph spectrum. On the one hand, copiotrophs are thought to be able to access a wider range of substrates (Saifuddin et al., 2019), yet, on the other hand, there is also evidence that oligotrophs are more nutritionally flexible (Upton and Nedwell, 1989). Besides the mixed results on substrate utilization, less is known about metabolite production among groups. Considering the importance of metabolites in mediating species interactions in microbial communities (Zengler and Zaramela, 2018; Niehaus et al., 2019) and contributing to persistent soil C (Liang et al., 2017; Lehmann et al., 2020), improving our understanding of how microbial life-history traits relate to metabolic activities is critical to understanding microbial contributions to soil C dynamics (Muscarella et al., 2020; Barnett et al., 2021).

Traditionally, microbial metabolic capacities are characterized by profiling the abilities of microorganisms to utilize a panel of individual C substrates, for example, using Biolog plates (Konopka et al., 1998). In these cases, the number of substrates utilized by each organism is used to estimate their resource niche width and the degree of overlapping growth-supporting substrates is used to estimate niche overlap for predicting resource competition between organisms (Wilson and Lindow, 1994; Dundore-Arias et al., 2019; Michalska-Smith et al., 2022). In natural environments (e.g., soils), microorganisms often encounter diverse substrates and can have different utilization strategies (Wang et al., 2019). Yet, relatively few studies have investigated microbial metabolic activities in mixtures of ecologically relevant substrates.

Recent advances in mass spectrometry-based metabolomics help fill this gap by enabling comprehensive analyses of chemically diverse samples (Bauermeister et al., 2022). By profiling changes of extracellular metabolites (“exometabolites”) in medium before and after cultivation (i.e., control vs. “spent” medium), we can characterize substrates that are preferentially depleted as well as metabolites that are secreted by individuals or communities of microorganisms (Silva and Northen, 2015; de Raad et al., 2022). Using an exometabolomics approach, we previously demonstrated that sympatric bacteria isolated from biocrusts have divergent substrate preferences (Baran et al., 2015). Furthermore, exometabolite profiles can be used to make inferences about metabolic interactions (e.g., metabolite competition or exchange) and facilitate the design of synthetic consortia (Erbilgin et al., 2017; Kosina et al., 2018).

Here, we aimed at exploring how metabolite consumption and production of bacteria differ among copiotrophic and oligotrophic life-history strategists and how the differences govern metabolic interactions between species. We selected bacteria from genera that were found to be among the most ubiquitous and abundant in soils, which belonged to the orders Micrococcales, Corynebacteriales, and Bacillales. Isolates were chosen to represent a contrast between species with low and high *rrn* copy numbers, which differed broadly according to several genomic traits (e.g., genome size, GC content, and prototrophy for biosynthetic pathways). We characterized exometabolite profiles of individual isolates grown in a defined medium containing a mixture of common water-soluble soil metabolites (Jenkins et al., 2017). By evaluating overlap in isolate substrate utilization profiles, we estimated the potential for competitive interactions among isolates, which was then tested by measuring pairwise interactions through sequential growth experiments. We predicted that *rrn* copy number would influence the metabolic properties and interactions between bacteria.

## Results

### Isolate Selection and Comparative Genomics

**FIGURE 1:**
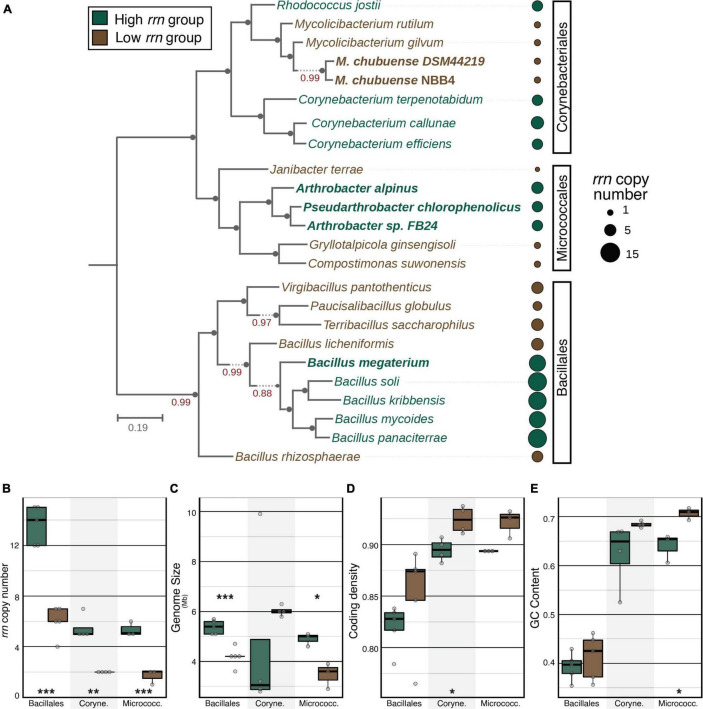
Phylogenetic tree and genomic features of 24 isolates. Detailed information is summarized in Supplementary Table 1. **(A)** A maximum-likelihood phylogenetic tree constructed from a multi-locus sequence alignment using “Insert Set of Genomes into Species Tree” in KBase. Bold isolates denote their genomes had 100% 16S rRNA similarity to an amplicon sequence variant detected in soil samples. For each phylogenetic order, tree leaves and annotation dots are colored based on order-specific *rrn* copy group. The size of each dot is scaled by absolute *rrn* copy number present in each genome. **(B)** Within each phylogenetic order, isolates from high vs. low *rrn* groups had significant differences in *rrn* copy number. High and low *rrn* copy groups also differed according to **(C)** genome size, **(D)** coding density, and **(E)** GC content. Asterisks denote statistically significant differences between high and low *rrn* copy groups within each phylogenetic order according to a Student’s *t*-test (*p ≤ 0.05, **p ≤ 0.01, and ***p ≤ 0.001).

We identified three bacterial species groups (phylotypes) based on their high relative abundance and widespread occurrence in a 16S rRNA gene-based survey of agricultural soils across the United States (Wilhelm et al., 2022a,b). Representative sequences for these phylotypes were classified to the genera *Arthrobacter*, *Bacillus*, and *Mycolicibacterium* and were detected in 91% ($\bar{x}$ = 1.2% of total reads), 65% (0.2%), and 61% (0.3%) of 778 samples, respectively. The *Arthrobacter* and *Bacillus* phylotypes were among those that rapidly responded (i.e., increased in relative abundance) in soils amended with dissolved organic C (xylose or glucose), while *Mycolicibacterium* did not (Barnett, 2021). The *Arthrobacter* and *Bacillus* phylotypes were also more abundant in agricultural soils than in less disturbed soils, such as old field or forest soils (Supplementary Figure 1). These observations led us to select members of these species groups as representatives of copiotrophic (*Arthrobacter* and *Bacillus*) and oligotrophic (*Mycolicibacterium*) soil populations. Isolate genomes that had 100% 16S rRNA similarity to the *Arthrobacter* and *Bacillus* groups had relatively high *rrn* copy number, while genomes matching the *Mycolicibacterium* group had relatively low *rrn* copy number (Figure 1A, isolates highlighted in bold; Supplementary Table 1). We expanded the collection of isolates to include an equal number of phylogenetically related isolates from neighboring families with contrasting *rrn* copy numbers (“phylo-pair”). A total of 24 isolates were selected, representing an even number of isolates with high and low *rrn* copy numbers within each of the three bacterial orders: Micrococcales, Corynebacteriales, and Bacillales (Figures 1A,B and Supplementary Table 1).

Several genomic properties differed between high and low *rrn* copy number groups. Bacillales and Micrococcales isolates with high *rrn* copy number had significantly larger genomes than low *rrn* copy pairs (Figure 1C). Within each phylogenetic order, low *rrn* copy genomes had consistently higher coding density and GC content than high *rrn* copy genomes, though these differences were only significant among Corynebacteriales (coding density; Figure 1D) and Micrococcales phylo-pairs (GC content; Figure 1E). Bacillales and Corynebacteriales isolates with low *rrn* copy numbers tended to be more auxotrophic than high *rrn* copy pairs, with the difference being significant for Corynebacteriales (Supplementary Figure 2).

### Isolate Growth Characteristics

We compared the growth characteristics of isolates among *rrn* copy and taxonomic groups by culturing individual strain in a soil defined medium (SDM). The SDM was previously constructed based on the water-soluble soil metabolite profiles and has been shown to support the growth of a diverse range of bacteria isolated from soil (Jenkins et al., 2017). Here, we modified the SDM composition to include common soil metabolites, root exudates, and essential cofactors, for a total of 89 organic compounds (Supplementary Table 2). Isolate growth rate [*F*_(2,13)_ = 4.4, *p* = 0.03] and doubling time [*F*_(2,13)_ = 8.1, *p* = 0.005] in the SDM differed primarily by taxonomic groups as determined by analysis of covariance (ANCOVA), with Corynebacteriales having the slowest rate (*p* = 0.01) and longest doubling time (*p* = 0.002; Supplementary Figures 3A,B). Both taxonomic [*F*_(2,13)_ = 14, *p* < 0.0001] and *rrn* copy groups [*F*_(1,13)_ = 8.2, *p* = 0.01] had significant effects on the carrying capacity, with carrying capacity being higher for isolates from the high *rrn* than low *rrn* copy group (Supplementary Figure 3C). When regressed against the absolute *rrn* copy number across isolates, growth rate was significantly positively correlated with *rrn* copy number (*R* = 0.53, *p* = 0.03), while doubling time (*R* = −0.50, *p* = 0.04) was negatively correlated with *rrn* copy number. Several isolates grew poorly in liquid SDM and were not included in downstream analyses for substrate utilization profiling (Supplementary Table 1).

### Isolate Substrate Utilization

**FIGURE 2:**
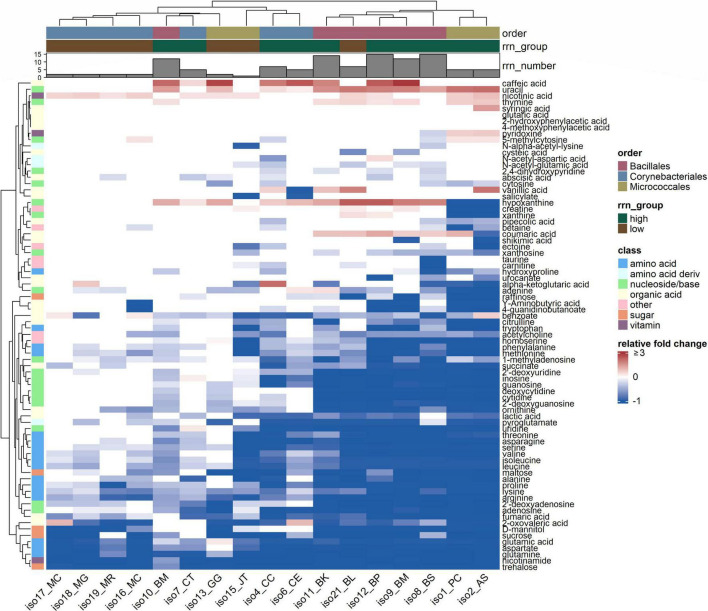
Relative fold changes of metabolite abundances in isolate spent media as compared with the soil defined medium control. Values are displayed as the mean of three replicates. A positive (red) or negative (blue) value indicates isolate production or depletion of that compound in the soil defined medium, respectively. For compound whose abundance did not differ significantly between spent and control media, relative fold change was 0 and shown in white (*p* > 0.05, one-way ANOVA with *post hoc* Dunnett’s test). Metabolites are annotated by compound class. Isolates are annotated by phylogenetic order, order-specific *rrn* copy group (i.e., high vs. low within each order), and absolute *rrn* copy number present in the genome.

To determine the depletion of SDM components by each isolate, we profiled isolate spent medium at the early stationary phase using liquid chromatography-mass spectrometry (LC-MS). In total, 78 out of the 89 compounds with equal molar concentrations in the SDM were tracked (Supplementary Table 3). Relative fold changes were calculated for compounds that had significantly different peak heights in the spent medium than in the uninoculated SDM control. Overall, isolates displayed differential utilization for the components of SDM (Figure 2). Each isolate depleted at least one substrate by over 97%. Isolate taxonomic group [pseudo *F*_(2,11)_ = 3.1, *p* = 0.009] and *rrn* copy group [pseudo *F*_(1,11)_ = 2.8, *p* = 0.04] each explained significant variation in substrate utilization patterns, accounting for 23 and 10% of the total variation, respectively (as determined by permutational multivariate analysis of variance, PERMANOVA). The interaction between *rrn* copy group and taxonomic group was significant, explaining an additional 17% of the variation [pseudo *F*_(2,11)_ = 2.3, *p* = 0.03].

**FIGURE 3:**
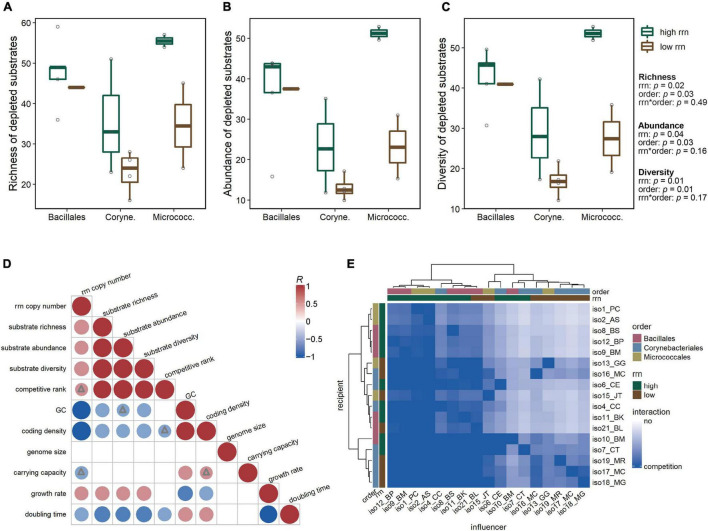
Substrate utilization and competition potential. Differences in substrate utilization among taxonomic and *rrn* copy groups reflected by **(A)** the richness, **(B)** abundance, and **(C)** diversity of substrates used by each isolate. All three substrate utilization metrics were significantly affected by *rrn* copy group and taxonomic group (ANCOVA, *p* < 0.05). **(D)** Pearson correlation coefficients (*R*) between isolate substrate utilization metrics, predicted competitive ranking, genomic features, and estimated growth parameters. Positive correlations are displayed in red and negative correlations in blue. Color intensity and size of the circles are proportional to the correlation coefficients. Circles without triangles represent significance level *p* < 0.05; triangles represent 0.05 < *p* < 0.1. For insignificant correlations (*p* > 0.1), the correlation coefficients are left blank. **(E)** Heatmap of directional pairwise competitive interaction strengths, predicted based on overlap in substrate utilization profiles. In heatmap color bar, label “no” represents no overlap in substrate use profiles, predicting no competition between isolates; darker blue color represents higher similarity in substrate use, predicting a higher potential that the influencer will compete with the recipient for resources.

To quantify differences in substrate utilization between groups, we analyzed the following metrics for each isolate: (1) the richness of substrates used (as the total number of substrates depleted), (2) the abundance of substrates used (as the sum of the percentages of depletion), and (3) the diversity of substrate utilization using Simpson’s diversity index (specifically the inverse of Simpson’s measure of concentration, which considers both richness and evenness, with a higher index indicating even utilization of multiple substrates and a lower index indicating strong preference for a limited number of substrates) (MacArthur, 1984; Zak et al., 1994). An ANCOVA revealed that both *rrn* copy and taxonomic groups had significant effects on the three metrics assessed (*p* < 0.05; Figures 3A–C). In particular, substrate richness [*F*_(1,13)_ = 6.9, *p* = 0.02], abundance [*F*_(1,13)_ = 5.2, *p* = 0.04], and diversity [*F*_(1,13)_ = 8.6, *p* = 0.01] were all significantly higher for isolates from the high *rrn* than the low *rrn* copy group. When regressed against the absolute *rrn* copy number across all isolates, the three metrics describing substrate utilization were all significantly positively correlated with *rrn* copy number (richness: *R* = 0.60; abundance: *R* = 0.52; diversity: *R* = 0.60; all *p* < 0.05; Figure 3D). Additionally, there were positive correlations between these metrics and the estimated growth rate (richness: *R* = 0.55; abundance: *R* = 0.54; diversity: *R* = 0.58; all *p* < 0.05; Figure 3D).

### Predicting Competitive Interactions From Substrate Utilization Patterns

Overlap in resource utilization can be used to estimate intensity of competition between organisms (Schoener, 1974). We predicted the potential strength of competition of each “influencer” isolate to another “recipient” isolate by calculating the directional overlap in their substrate use profiles (refer to the Section “Materials and Methods,” Equation 1) (MacArthur and Levins, 1967; MacArthur, 1984). We found that the Micrococcales and Bacillales isolates appeared to have a higher potential to compete for substrates than the Corynebacteriales isolates, as indicated by the clustering of influencer isolates with higher predicted competition strengths (Figure 3E, top dendrogram). Additionally, isolates from the high *rrn* group tended to have a higher potential for resource competition than low *rrn* isolates.

We further estimated the competitive ranking of each isolate based on the predicted competition strengths (refer to the Section “Materials and Methods,” Equation 2) (Carrara et al., 2015), where the higher the rank, the greater the potential for competitive interactions with other isolates. We found the predicted competitive rank was significantly positively correlated with the three substrate utilization metrics (richness: *R* = 0.89; abundance: *R* = 0.97; diversity = 0.92; all *p* < 0.001) and negatively correlated with isolate doubling time (*R* = −0.52, *p* = 0.05; Figure 3D). The competitive ranking was also positively correlated with isolate *rrn* copy number (*R* = 0.44, *p* = 0.08; Figure 3D).

### Testing Pairwise Interactions Using Spent Media

To experimentally evaluate predicted interactions, we selected eight isolates representing different clusters in substrate utilization profiles (Figure 2) and potential competition strengths (Figure 3E). The eight representatives consisted of four Micrococcales and four Corynebacteriales, each set comprising two low and two high *rrn* isolates (Supplementary Table 1). As only one out of five isolates from the low *rrn* Bacillales group grew during the exometabolomic experiment, Bacillales isolates were excluded from this analysis to avoid unbalanced comparisons. We characterized directional pairwise interactions by conducting a sequential growth screening of each isolate using spent media from all of the other isolates (Biggs et al., 2017; Ratzke et al., 2020). Specifically, spent medium from the first isolate (influencer) was collected and used to culture a second isolate (recipient). Growth was assessed with two metrics, namely, final optical density (OD) and cumulative respiration of the culture. The effect of the influencer on the recipient (i.e., interaction strength) was determined as the relative fold change of recipient’s growth in the influencer’s spent medium as compared with in SDM (refer to the Section “Materials and Methods,” Equation 3). A negative or positive value indicates a negative (e.g., competitive) or positive (e.g., facilitative) effect of the influencer isolate on the recipient isolate, respectively.

**FIGURE 4:**
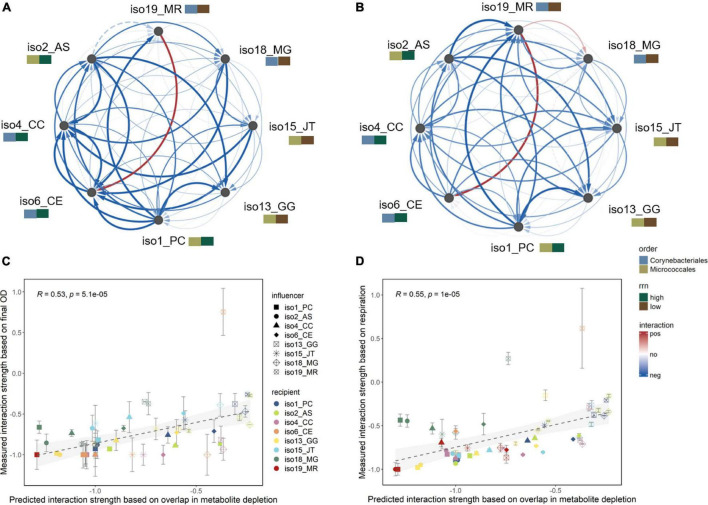
Directional pairwise interaction strengths. Measured interaction strengths from sequential growth experiments based on **(A)** final OD and **(B)** cumulative CO_2_ respiration results, shown as directed networks visualized in Cytoscape. Isolates are annotated by their phylogenetic order and order-specific *rrn* groups. Edge width and color correspond to mean relative fold change of the growth of recipient isolate (target node) in the spent medium of influencer isolate (source node) as compared with soil defined medium (*n* = 3). Red or blue represents increased or decreased growth, indicating positive or negative interaction, respectively. Insignificant interactions (*p* > 0.05, one-way ANOVA with *post hoc* Dunnett’s test) are shown in dashed edges. Results of individual replicates and correlation between the two interaction measures are shown in Supplementary Figure 4. Linear correlations between the predicted (Figure 3E) and measured significant interaction strengths based on **(C)** final OD and **(D)** cumulative respiration. Error bars represent standard deviations (*n* = 3). Correlation results for each individual recipient isolate are shown in Supplementary Figures 6,7.

Interactions were mostly negative, where recipient isolates had decreased growth (lower final OD or reduced CO_2_ respiration) in the influencer’s spent medium than in SDM (Figures 4A,B). Positive interactions were also observed, including the increased growth of *Corynebacterium efficiens* (iso6) in the spent medium of *Mycolicibacterium rutilum* (iso19). *Mycolicibacterium gilvum* (iso18) also had increased respiration when grown in iso19’s spent medium, although the final OD was lower. Still, the interaction strengths calculated using the two metrics (OD and respiration) were positively correlated (*R* = 0.54, *p* < 0.001; Supplementary Figure 4). The average interaction strengths were affected by the *rrn* copy group, but not the taxonomic group, of the influencer isolates, with influencers from the high *rrn* group having greater negative effects on recipients than influencers from the low *rrn* group [OD: *F*_(1,5)_ = 5.5, *p* = 0.07; respiration: *F*_(1,5)_ = 6.8, *p* = 0.05; ANCOVA; Supplementary Figure 5].

The measured interaction strengths were significantly positively correlated with those predicted based on substrate use overlap (OD: *R* = 0.53; respiration: *R* = 0.55; both *p* < 0.001; Figures 4C,D). The correlations were particularly strong for isolates that mainly experienced negative interactions (Supplementary Figures 6,7). This suggests that substrate competition was a dominant mechanism for most interacting pairs.

### Untargeted Metabolite Profiling of Spent Media

To further explore interaction mechanisms, especially the potential role of metabolic secretion and exchange, we conducted an untargeted exometabolite profiling of spent media before and after the sequential growth experiments. Initial untargeted analysis detected a list of LC-MS features, corresponding to specific mass-to-charge ratio (*m/z*) and retention time combinations (Supplementary Table 4). Only the features that were identified to have significantly higher abundances in samples than background were used for downstream analysis (described in detail in the Section “Materials and Methods”). We searched for features that were enriched in each isolate’s spent medium by comparing feature peak heights between spent medium and SDM control (Supplementary Table 5). On average, 10.4% (±3.0%, standard deviation) of features had significantly higher abundances in spent medium than in SDM, indicating metabolite production (Supplementary Figure 8). The percentage of features with increased abundances did not significantly differ by isolate *rrn* copy or taxonomic group (ANCOVA, *p* > 0.05; Supplementary Figure 8).

**FIGURE 5:**
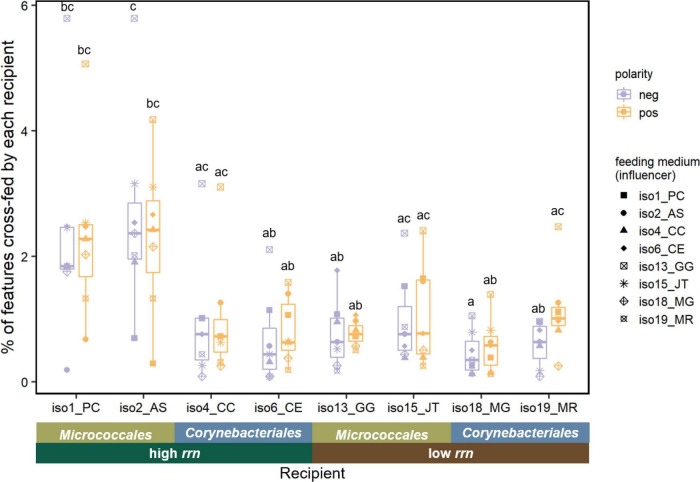
Percentage of LC-MS features cross-fed by each recipient isolate. Significant differences denoted using letters (*p* < 0.05, one-way ANOVA with *post hoc* Tukey HSD test). *n* = 7 for each recipient isolate under each ionization mode. Each data point represents the percentage of positive or negative features depleted by a recipient isolate grown on the spent medium of an influencer (corresponding to different shapes listed in the legend). An ANCOVA revealed that taxonomic group and its interaction with *rrn* copy group both had significant effects on the percentage of cross-feeding features depleted by the recipient isolate, under both polarities as well as the average result (*p* < 0.05). Feature count results can be found in Supplementary Table 5.

After culturing a recipient isolate in the spent medium of the influencer isolate, the resulting “double spent medium” was compared with the initial spent medium to identify metabolites significantly changed by the recipient isolate (Supplementary Table 5). For the two pairs of isolates showing positive interactions (Figure 4), 4 and 6 features displayed a pattern of cross-feeding for the iso6-iso19 pair and iso18-iso19 pair, respectively. Specifically, the relative abundance of these features increased in the spent medium of *M. rutilum* (iso19) compared with SDM, and then decreased after using this spent medium to grow either *C. efficiens* (iso6) or *M. gilvum* (iso18). This suggests the ability of these two isolates to consume iso19’s metabolic secretion, which could have contributed to their increased growth. Among all isolates, we found that on average 1.2% (±0.7%, standard deviation) of features displayed a cross-feeding pattern (Figure 5). Specifically, the average percentage of features cross-fed by each recipient isolate was significantly affected by both taxonomic group [*F*_(1,4)_ = 74, *p* = 0.001] and its interaction with *rrn* copy group [*F*_(1,4)_ = 27, *p* = 0.006; ANCOVA].

Putative annotations for significant features were obtained by comparing *m/z* and retention time to an in-house database of compound standards. Tandem mass spectrometry (MS/MS) fragmentation spectra were queried against a spectral library using the Global Natural Products Social Molecular Networking (GNPS) tool (Wang et al., 2016). Features with matching *m/z*, retention time, and MS/MS spectra were annotated (Supplementary Table 6). Relative fold changes were calculated for these metabolites and visualized to show whether they were depleted or produced by the influencer and recipient isolates during the sequential growth experiments (Supplementary Figures 9,10).

## Discussion

### Genomic Signatures of Life-History Strategies

We characterized the metabolic activities and interactions of bacteria representing copiotrophic and oligotrophic life-history strategies. Isolates were chosen based on three phylotypes that were found to be ubiquitous and abundant in soils, exhibit different responses to resource availability and disturbance, and differ in *rrn* copy number. We used *rrn* copy number as a proxy for bacterial strategies because copiotrophs (with traits typical of zymogenous or *r*-selected strategists) often contain higher *rrn* copy numbers, while oligotrophs (with traits typical of autochthonous or *K*-selected strategists) often contain lower *rrn* copy numbers (Fierer et al., 2007; Prosser et al., 2007). High *rrn* copy number is positively correlated with growth rate (Roller et al., 2016; Pold et al., 2020), and this relationship was evident among the isolates studied here (Figure 3D). Bacteria adapted for rapid growth in soil must produce ribosomes quickly to meet demand for protein synthesis during the transient periods in soil that support high growth rates. This rapid ramp up in ribosome copies is promoted by having high *rrn* copy number (Polz and Cordero, 2016; Roller et al., 2016).

Several genomic properties differed between high and low *rrn* copy phylo-pairs. Genomes with low *rrn* copy number tended to have higher coding density and GC content, and smaller genomes that encoded fewer biosynthetic pathways (Figure 1 and Supplementary Figure 2). The positive correlation between genome size and *rrn* copy number has previously been shown (Roller et al., 2016). The relationships between *rrn* copy number and genome GC content and coding density identified for the strains analyzed here have not previously been described. In marine oligotrophs, genome streamlining corresponded with smaller genome size, higher coding density, and lower GC content (Giovannoni et al., 2014). Our results for the soil bacterial isolates studied here are consistent with trends in genome size and coding density, but not GC content, which might suggest differences shaping the genomes of bacteria adapted to marine vs. soil habitats. Still, these observations show that *rrn* copy number is associated with broad changes in genome composition, suggesting that this trait is correlated with fundamental adaptive differences among strains.

### Isolate *rrn* Copy Number Correlates With Substrate Utilization

Strains from the high *rrn* group depleted a greater diversity and amount of metabolites than low *rrn* strains (Figures 3A–C), when grown in a chemically defined medium containing a mixture of common soil metabolites (Figure 2). Previously, the ability to use simple carbon substrates has been shown to be a shallowly phylogenetically conserved trait (Martiny et al., 2015). Positive correlations were observed between phylogenetic distance and substrate use dissimilarity, indicating that more closely related bacteria had more similar resource use profiles (Schlatter et al., 2013; Dolan et al., 2017). Here, we found substrate utilization patterns not only varied among taxonomic groups, in agreement with previous findings, but also differed between *rrn* copy groups. Bacteria with large genomes have been shown to have a tendency to occupy a broader range of soil habitats (Barberan et al., 2014) and considered to be able to access a wider range of C substrates (Saifuddin et al., 2019). Furthermore, copiotrophic bacteria with higher *rrn* copy numbers have high numbers of transporter genes (Lauro et al., 2009). In addition, bacteria found to be generalists had higher *rrn* copy numbers and more metabolic genes than bacteria found to be specialists (Dal Bello et al., 2021). Our experiments show that *rrn* copy number was positively correlated with the richness, abundance, and diversity of metabolites consumed (Figure 3D), suggesting high *rrn* copy number associates more with a generalist resource use style, while low *rrn* copy number associates with a higher degree of specialization. However, these results contrast with previous experiments using single substrate profiling that have found oligotrophic bacteria to utilize a higher number of organic substrates than copiotrophs (Upton and Nedwell, 1989), as supported by theoretical prediction (Button, 1994). These mixed results highlight the complexity of comparing resource use capacity across the copiotroph–oligotroph spectrum, which depends on factors including the concentration of limiting nutrient and the dynamics of growth (batch vs. steady state). Experiments performed in culture must always be interpreted with care because the determinants of fitness in culture might not represent well the determinants of fitness *in situ*. Moreover, as a study of this nature cannot capture the full range of bacterial genomic or ecophysiological diversity, extrapolation of the results should be treated with caution. Still, the evidence from our study fits within a long line of evidence showing generalizable differences according to *rrn* copy number and other genomic traits indicative of life-history strategies.

### Substrate Use Overlap Predicts Interactions Dominated by Resource Competition

We predicted that isolates from different *rrn* groups would differ in their potential for resource competition since substrate utilization correlated with *rrn* copy number. We estimated the potential strength of competition by determining the extent of overlap in isolate substrate use profiles (Figure 3E). Our prediction was built on previous work using the number of overlapping substrates to infer competition (Wilson and Lindow, 1994; Kosina et al., 2018) and further considered the varying degree of utilization to capture the differential substrate preference by each isolate. We found that the predicted competitive rank of each isolate was positively correlated with substrate utilization and *rrn* copy number (Figure 3D).

The predicted competitive interactions correlated with the measured interactions that were dominated by negative interactions (Figure 4). Isolates from the high *rrn* group had greater negative effects on others than isolates from the low *rrn* group as predicted (Supplementary Figure 5). Previously using a similar sequential growth approach, it was found that 75% of the measured interactions were in qualitative agreement with the sign of interaction coefficients estimated using generalized Lotka-Volterra models (Venturelli et al., 2018). Here, our experiments showed that 105 out of 108 significant interactions were negative, in agreement with predictions based on overlap in substrate utilization patterns (Figure 4). More quantitatively, the measured interaction strengths were positively correlated with the predicted competition strengths. These results suggest that resource competition was a major force causing negative interactions among the isolates tested under experimental conditions of this study. By profiling spent medium metabolites during the sequential growth experiment, we identified substrates that were sequentially depleted by both isolates, providing evidence for competition (Supplementary Figures 9,10). Previously, prevalence of negative competitive interactions has also been reported for gut microbiota (Biggs et al., 2017) and bacteria isolated from tree-hole aquatic habitats (Foster and Bell, 2012).

The significant correlations between the measured and predicted interaction outcomes also indicate that isolate exometabolite profiling coupled with measure of substrate use overlap can be a powerful tool for predicting pairwise interactions, which can be used to infer behaviors of more complex communities (Friedman et al., 2017; Venturelli et al., 2018). Still, it is noteworthy that correlations were stronger for some recipient isolates and weaker for others (Supplementary Figures 6,7). This could be explained, in part, if substrate use efficiency varies among substrates (Saifuddin et al., 2019; Muscarella et al., 2020). Therefore, the same degree of overlap for two substrates may not necessarily translate to the same degree of growth inhibition for each organism. In our prediction, we treated each substrate as equally important and independent (Michalska-Smith et al., 2022). One future direction to improve prediction would be to develop weighting factors to take into account the varying importance and uniqueness of each metabolite to the specific bacteria concerned (Colwell and Futuyma, 1971). However, the feasibility will depend on improved biochemical knowledge of metabolite functions in diverse bacteria.

### Rare Positive Interactions Likely Caused by Metabolic Cross-Feeding

Previous work using genome-scale metabolic modeling has predicted that there are a diverse range of metabolic byproducts, such as organic acids and carbohydrates, that can be secreted by microorganisms and enable beneficial inter-species interactions (Zelezniak et al., 2015; Pacheco et al., 2019). However, empirical studies to validate such interactions are limited. Here, we detected on average 10.4% of LC-MS features had increased abundances after growing each isolate individually in SDM. We had expected to see high *rrn* isolates to release more byproducts since they are associated with fast but inefficient growth, which might promote secretion of metabolic intermediates (Polz and Cordero, 2016). However, we did not find the number of released features significantly affected by *rrn* copy or taxonomic group (Supplementary Figure 8). However, the number of released features that were then consumed (cross-fed) by the recipient isolate varied significantly with taxonomic group and its interaction with *rrn* copy group (Figure 5). The two high *rrn* Micrococcales isolates, *Pseudarthrobacter chlorophenolicus* (iso1) and *Arthrobacter* sp. (iso2), appeared to consume higher numbers of features released by other isolates, yet they did not show significantly higher growth in spent media. Similar trends were reported for pairwise interactions in gut microbiota, where ∼2% of metabolite comparisons indicated potential cross-feeding but no net growth promotion was observed (Biggs et al., 2017). This result could be because cross-feeding was not sufficient to offset the growth reduction due to depletion of other limiting resources in spent media or because the benefits of cross-feeding were masked in batch culture grown on artificial media.

The three positive interaction cases that we indeed observed could be attributed to the exchange of secreted metabolites. The positive interactions were unidirectional and the low *rrn* strain *M. rutilum* (iso19) was the influencer. Both *M. rutilum* and both recipient strains were members of the Corynebacteriales group (Figure 4). This finding is consistent with prior predictions using genome-scale metabolic models that unidirectional positive interactions are more common than bidirectional positive interactions (Freilich et al., 2011; Pacheco et al., 2019). Because only a relatively limited number of features were annotated using the untargeted metabolomics pipeline, the exact cross-fed metabolites that could have contributed to these positive interactions could not be identified at this time.

## Conclusion

We found that substrate utilization and competition among common soil bacteria varied with *rrn* copy number. Among the isolates studied here, isolates with high *rrn* copy number tended to grow faster, consume more substrates, and have a higher potential for competitive interactions than isolates with low *rrn* copy number. Overlap in substrate utilization patterns predicted interactions that were dominated by negative competition, with relatively rare positive interactions likely caused by cross-feeding of metabolic byproducts. Coupling analysis of *rrn* copy number with exometabolite profiling of individual isolates could aid the design of synthetic microbial consortia and help explain interaction patterns in complex natural communities. In the longer term, the scientific exploration of relationships between bacterial life-history strategies, genomic traits, and metabolic activities can advance the understanding of microbial metabolic control of soil C cycling.

## Materials and Methods

### Isolate Selection and Genomic Analysis

We curated a collection of soil bacteria (*n* = 24, Supplementary Table 1) that included at least three representatives for each species group of copiotrophs (*Arthrobacter* and *Bacillus*) and oligotrophs (*Mycolicibacterium*), as well as an equal number of phylogenetically related isolates that had contrasting *rrn* copy number (phylo-pair). Specifically, phylo-pairs were selected by querying the IMG-ER portal (Markowitz et al., 2007) to identify publicly available genomes classified to Micrococcales, Corynebacteriales, and Bacillales that met the following criteria: (1) fewer than 10 contigs and/or raw sequencing data available to validate *rrn* copy number abundance, (2) from the same or closely related family, (3) maximally contrasting *rrn* copy numbers among closest relatives, (4) available in culture collections, and (5) isolated from soil. If more than four genomes met these criteria, those with the highest quality genome assembly were selected. Genomes were downloaded from the NCBI refseq genome database in January 2019. Twenty-two bacterial strains were sourced from the Leibniz Institute DSMZ—German Collection of Microorganisms and Cell Cultures, and two from the Bacillus Genetic Stock Center and a researcher, with detailed information summarized in Supplementary Table 1.

The *rrn* copy number was determined using two complementary approaches, namely, (1) recovering all 16S rRNA genes using *barrnap* (v. 0.4.2^1^), using hidden Markov model, then manually correcting for genes that were incorrectly duplicated by errors in assembly, and (2) relative read depth between the 16S rRNA gene and putative single copy genes identified by *BUSCO* (v. 4.1.2) (Simão et al., 2015). The *rrn* copy values obtained were comparable to those listed on the IMG-ER, but manually validated. Auxotrophies were determined for each representative genome and phylobin using “Genome-Enabled Metabolic Models” (Henry et al., 2010) in KBase (Arkin et al., 2018). The phylogeny of strains was determined from a maximum-likelihood tree based on a multi-locus sequence alignment (MLSA) using “Insert Set of Genomes into Species Tree” (v. 2.1.10) in KBase.

### Isolate Growth Measurement and Exometabolomic Sampling

Soil defined media (SDM) was constructed as previously described (Jenkins et al., 2017) with slight modifications. In total, 89 common soil metabolites were added at equimolar concentration (20 μM) and mixed with KH_2_PO_4_ (0.6 g L^–1^), NH_4_Cl (1.5 g L^–1^), Wolfe’s vitamin and Wolfe’s mineral solutions, each at 1X concentration (Supplementary Table 2).

Growth curves were obtained on a BioLector microbioreactor (Beckman Coulter GmbH, Baesweiler, Germany) using 48-well microtiter plates. Isolates were revived from glycerol stocks on TSB plates and then colonies were transferred to SDM plates (SDM with 1.5% Noble agar). Starter liquid cultures were created by inoculating 3 ml liquid SDM with a single colony from SDM plates into 5 ml clear culture tubes and cultured at 28°C. Each isolate was diluted from starter cultures to an OD at 600 nm (OD_600_) of 0.02 in a final volume of 1.5 ml liquid SDM per well. The plate was shaken at 800 rpm and incubated at 28°C with readings taken every 20 min. Growth curves were fit using the R package *growthcurver* (v. 0.3.1) (Sprouffske and Wagner, 2016) to estimate doubling time, maximum growth rate, and carrying capacity.

For exometabolomics, starter cultures and plates were prepared similarly as for growth curves. In total, two plates were used for exometabolomics. The following two sets of uninoculated medium controls were included on each plate to account for any non-biological metabolite degradation during the incubation period: an early control collected at the first time point (when fast-growing isolates were sampled) and a late control collected at the last time point (when slow-growing isolates were sampled). Each isolate and uninoculated medium control were run in triplicate. Samples were collected for each isolate at the estimated early stationary phase. At sampling, the entire volume was collected for each well and centrifuged at 5,000 × *g* for 10 min. Supernatants were transferred to 1.5 ml tubes and stored at −80°C for LC-MS analysis (described in detail below).

To predict potential for resource competition, we calculated the predicted interaction strength (*PIS*_ri_) of influencer strain (*i*) to recipient strain (*r*) based on the directional overlap in isolate substrate utilization profiles (MacArthur and Levins, 1967; MacArthur, 1984):


(1) $${\mathrm{PIS}}_{\mathrm{ri}}=-\frac{\sum_{m}p_{m,r}\,p_{m,i}}{\sum_{m}p_{m,r}^{2}},$$

Where *p*_m,r_ and *p*_m,i_ are the percentages of metabolite *m* significantly depleted by strain *r* and *i*, respectively, when individually grown in SDM containing 89 metabolites of equimolar concentration. Negative sign denotes the predicted interaction is negative as a result of competition.

The competitive rank of isolate *r* (*R*_*r*_) was then estimated by summing the predicted negative effects of all the other isolates on the focal isolate *r* and subtracting the negative effects of the focal isolate *r* on all the other isolates (Carrara et al., 2015):


(2) $$R_{r}=\sum_{i}\left({\mathrm{PIS}}_{\mathrm{ri}}-{\mathrm{PIS}}_{\mathrm{ir}}\right),$$

The bigger *R*_r_ is, the greater the potential does isolate *r* have for competitive interactions with other isolates.

### Sequential Growth Experiment

Spent media were prepared by growing each individual isolate in fresh SDM for 48 h and then centrifuging at 5,000 × *g* for 10 min to pellet cells and collect supernatant. The supernatant was filter sterilized using a 0.22 μm membrane and supplemented with 2% (v/v) 50X SDM base solution (only containing KH_2_PO_4_, NH_4_Cl, Wolfe’s vitamin and Wolfe’s mineral solutions without metabolites) to replenish these other nutrients and prevent them from being growth limiting factors. The resulting spent media from the first isolate (influencer) were used to culture a second isolate (recipient).

Starter cultures of recipient isolates in liquid SDM were centrifuged, cell pellets resuspended in 1X SDM base solution to an OD_600_ of 0.5, and diluted into the spent media of seven other isolates to a final OD_600_ of 0.01 to start the sequential growth experiment. Concentrated cultures were also diluted into SDM that was similarly amended with 2% (v/v) 50X SDM base solution to serve as controls for growth comparison. Uninoculated SDM and uninoculated, filter-sterilized initial spent media were also included as abiotic controls. Each isolate and uninoculated control were prepared in triplicate. OD_600_ readings were taken every 30 min with orbital mixing at 28°C for 48 h using a Biotek Synergy HT microplate reader (Biotek Instruments, Winooski, VT, United States). Parallel plates were setup to measure respired CO_2_ using the Microresp method (Campbell et al., 2003; Foster and Bell, 2012). Briefly, a 96-well microplate containing cresol red indicator gel was placed on top of a two-way rubber sealing mat that connected each well to a deep-well plate containing samples below. A metal clamp was used to hold the plates together and ensure an even seal. This design allowed for the movement of respired CO_2_ gas produced by each isolate to react with the indicator gel. Cumulative CO_2_ respiration was estimated as the difference between the initial and final absorbance at 570 nm (A_570_) measured of the indicator plate after baseline correction using uninoculated medium controls. At the end of growth, cultures were centrifuged and supernatants were collected and stored at −80°C for LC-MS analysis.

Relative change in growth was calculated as the measured interaction strength (*MIS*_ri_) of influencer strain (*i*) to recipient strain (*r*):


(3) $${\mathrm{MIS}}_{\mathrm{ri}}=\frac{{\mathrm{Growth}}_{r,i}}{{\mathrm{Growth}}_{r,\mathrm{SDM}}}-1,$$

Where *Growth*_r,i_ and *Growth*_*r,SDM*_ represent the growth of the recipient in the influencer’s spent medium or in SDM, respectively. A negative or positive *MIS*_ri_ indicates a negative (e.g., competitive) or positive (e.g., facilitative) effect of the influencer on the recipient, respectively. By the definition, *MIS*_ri_ is ≥ −1, with *MIS*_ri_ = −1 when the recipient strain did not grow at all in the influencer’s spent medium. Directed interaction networks were visualized in Cytoscape (Shannon et al., 2003).

### Extraction and LC-MS Analysis

Exometabolomic samples were lyophilized overnight and resuspended in 150 μl methanol containing internal standards (Supplementary Table 7). The resuspended samples were vortexed, sonicated in ice water bath for 15 min, centrifuged at 10,000 × *g* for 5 min at 4°C, supernatants filtered through 0.2 μm modified nylon membrane centrifugal filters, and filtrates collected for analysis. Samples were analyzed using normal-phase LC-MS using a HILIC-Z column (150 mm × 2.1 mm, 2.7 μm, 120Å, Agilent Technologies, Santa Clara, CA, United States) on an Agilent 1290 Infinity UHPLC. MS data were collected on a Thermo QExactive (Thermo Fisher Scientific, Waltham, MA, United States) and MS/MS data collected using collisions energies of 10–40 eV. Detailed instrument parameters are described in Supplementary Table 7. Each sample was analyzed in both positive and negative ionization modes. Sample injection order was randomized, and an injection blank of methanol only was run between each sample. Internal standards were included in all samples during the extraction process, and external standards were injected every 10 samples for quality control purposes.

### Metabolomics Data Analysis

Soil defined medium metabolites were identified through a targeted analysis pipeline using Metabolite Atlas^2^ (Yao et al., 2015) with extracted ion chromatograms and peak heights obtained for each metabolite using in-house Python scripts. Metabolite identifications were verified with authentic chemical standards and validated based on three metrics (matching *m/z*, retention time, and MS/MS fragmentation spectra). Data from internal standards and quality control samples were analyzed to ensure consistent peak heights and retention times. One-way ANOVA with *post hoc* Dunnett’s test was conducted to compare significant differences in metabolite peak heights between spent and control media (*n* = 3; raw data in Supplementary Table 3). For metabolite whose abundance did not differ in the spent from control media (*p* > 0.05), relative fold change was 0. For metabolite that had significantly different abundance (*p* < 0.05), relative fold change was calculated by dividing its peak height in isolate’s spent medium by peak height in the control and subtracting 1. A negative or positive value indicates depletion or production of that compound by the corresponding isolate.

Untargeted analysis of LC-MS data was performed similarly to previously described (Brisson et al., 2021). MZmine (Katajamaa et al., 2006; Pluskal et al., 2010) was used to detect features corresponding to specific *m/z* and retention time values. Samples were split into two sets and analyzed separately. Initial untargeted analysis detected 4,760 and 3,443 features for the two sets, respectively (Supplementary Table 4). Features with higher abundances in samples than background were identified by comparing feature peak heights in samples with peak heights in the extraction controls using one-way ANOVA with *post hoc* Dunnett’s test (*n* = 3). In total, 3,639 (2,063 positive and 1,576 negative) and 2,719 (1,579 positive and 1,140 negative) features were determined for the two sets to have higher abundances in at least one sample group than background (*p* < 0.05) and kept for downstream analysis. Significant changes in feature abundances between spent medium samples and corresponding medium controls were examined similarly as described above in targeted analysis. For features determined to be having significantly different abundances in spent than in control media, putative annotations were obtained by comparing *m/z* and retention time of standards analyzed using the same LC-MS method on the same instrument in our laboratory. MS/MS spectra were queried against the Berkeley Lab spectral library using GNPS (Wang et al., 2016).

## Data Availability Statement

The original contributions presented in this study are included in the article/Supplementary Material, further inquiries can be directed to the corresponding author.

## Author Contributions

YW, RW, TS, DB, and TN designed the study. YW, RW, and TS performed the experiments and analyzed the data. AS and PA contributed to the data analysis and interpretation. TS, AG, and SK acquired the LS-MS data. SK and BB advised the LC-MS data analysis. YW drafted the manuscript with input from RW, TS, and TN. All authors revised the manuscript and approved the final version for publication.

## Conflict of Interest

The authors declare that the research was conducted in the absence of any commercial or financial relationships that could be construed as a potential conflict of interest.

## Publisher’s Note

All claims expressed in this article are solely those of the authors and do not necessarily represent those of their affiliated organizations, or those of the publisher, the editors and the reviewers. Any product that may be evaluated in this article, or claim that may be made by its manufacturer, is not guaranteed or endorsed by the publisher.

## Footnotes

1
https://github.com/tseemann/barrnap

2
https://github.com/biorack/metatlas
