# Supplementary material for: Substrate Utilization and Competitive Interactions Among Soil Bacteria Vary With Life-History Strategies
Source: Front Microbiol. 2022 Jun 9;13:914472. doi: 10.3389/fmicb.2022.914472 (PMC9225577; doi:10.3389/fmicb.2022.914472)
Supplement: Supplementary file 1 [file Data_Sheet_1.ZIP › Supplementary figures.pdf]

Supplementary Material

for

Substrate Utilization and Competitive Interactions Among Soil Bacteria Vary with Life-History Strategies

Ying Wang[1], Roland C. Wilhelm[2], Tami L. Swenson[1], Anita Silver[1], Peter F. Andeer[1], Amber Golini[1], Suzanne M. Kosina[1], Benjamin P. Bowen[1,3], Daniel H. Buckley[2,4], Trent R. Northen[1,3*]

[1] Environmental Genomics and Systems Biology Division, Lawrence Berkeley National Laboratory, Berkeley, CA, United States

[2] School of Integrative Plant Science, Cornell University, Ithaca, NY, United States

[3] Joint Genome Institute, Lawrence Berkeley National Laboratory, Berkeley, CA, United States

[4] Department of Microbiology, Cornell University, Ithaca, NY, United States

*** Correspondence:**
Trent R. Northen: TRNorthen@lbl.gov

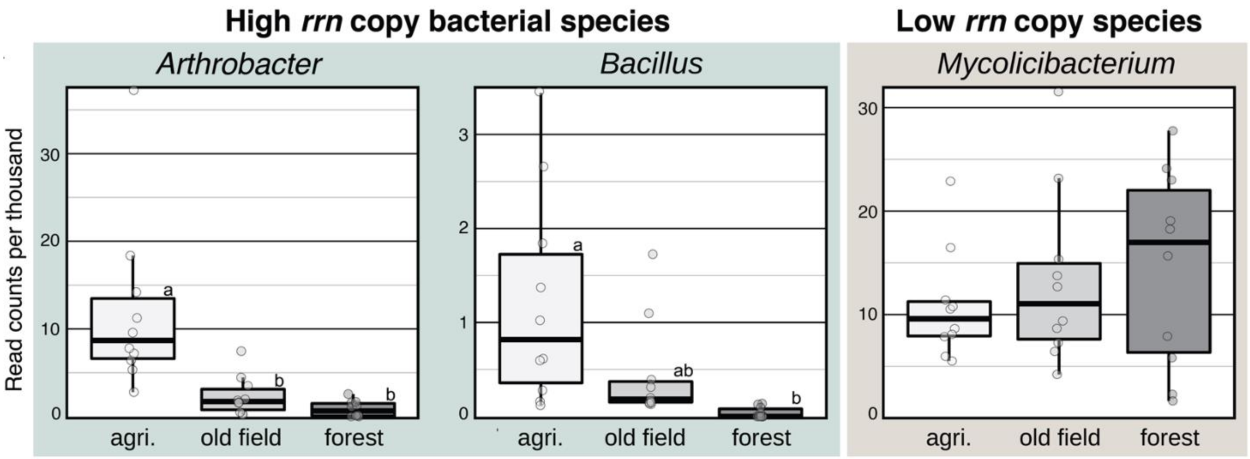

Supplementary Figure 1. Supporting evidence used to inform the selection of isolates for our study. 16S rRNA amplicon-based profiles of bacterial species with higher (*Arthrobacter* and *Bacillus*) *rrn* copy numbers had higher relative abundances in agricultural soil than in old field and forest soils. Lettering denotes significant differences in relative abundances according to pairwise Kruskal-Wallis testing ($p < 0.05$).

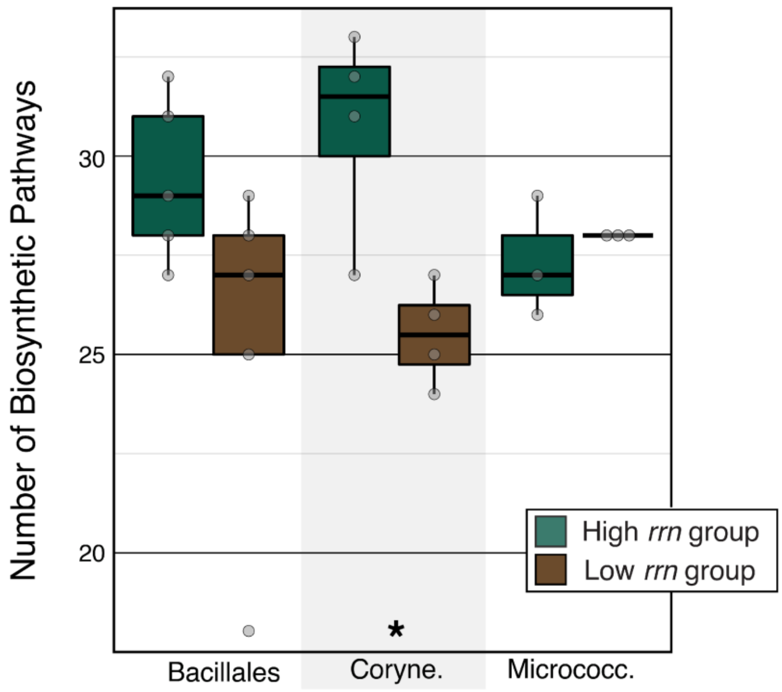

Supplementary Figure 2. Comparative genomics revealed differences in the number of biosynthetic pathways for essential metabolites (amino acids and co-factors) encoded by high and low *rrn* copy group isolates. Asterisk denotes significant difference between high and low *rrn* copy groups within Corynebacteriales according to a Student's *t*-test (* $p < 0.05$).

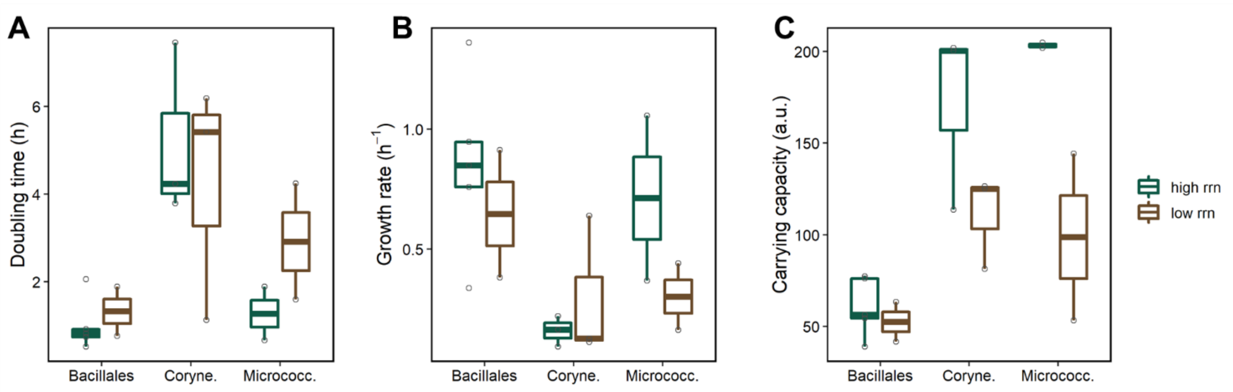

Supplementary Figure 3. Growth characteristics of isolates in the soil defined medium determined using a BioLector microbioreactor and shown for **(A)** doubling time, **(B)** maximum growth rate, and **(C)** carrying capacity that were modeled using the R package *growthcurver* (v. 0.3.1). In **(C)**, carrying capacity biomass is based on light scattering with an arbitrary unit as output provided by the BioLector. (n = 2 ~ 5; some isolates grew poorly in the defined medium so no growth parameters were estimated.)

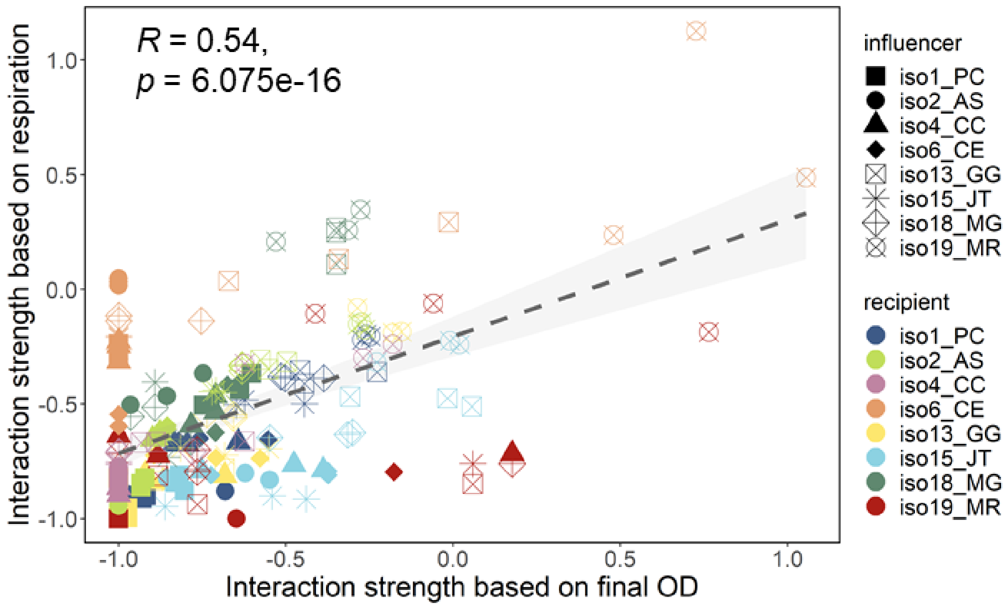

Supplementary Figure 4. Linear correlation between interaction strengths based on final OD and cumulative CO_2 respiration measured from sequential growth experiments. Data are shown for individual replicate results (n = 3).

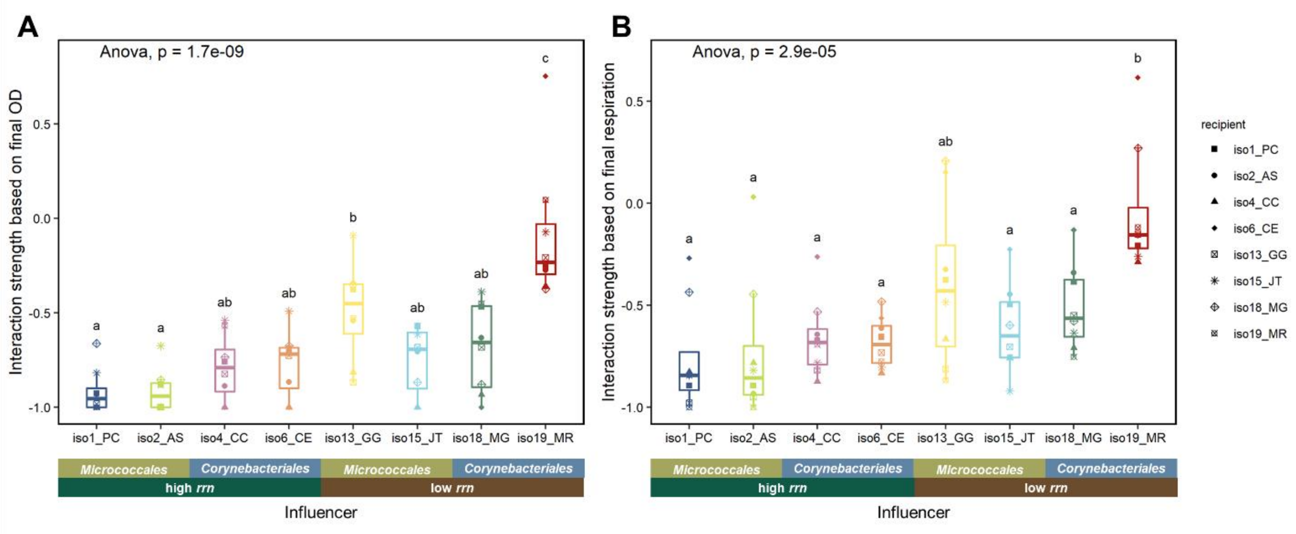

Supplementary Figure 5. Average interaction strengths by each influencer isolate, measured from sequential growth experiments based on **(A)** final OD and **(B)** cumulative CO_2 respiration results. Each point represents the mean effects of that influencer on a recipient (corresponding to different shapes listed in the legend; n = 3). Significant differences among influencers denoted using letters ($p < 0.05$, one-way ANOVA with post-hoc Tukey HSD Test). An ANCOVA revealed that influencer's average interaction strength differed by *rrn* copy group, with influencers from the high *rrn* group having greater negative effects on recipients than influencers from the low *rrn* group (OD: $F_{1,5} = 5.5$, $p = 0.07$; respiration: $F_{1,5} = 6.8$, $p = 0.05$).

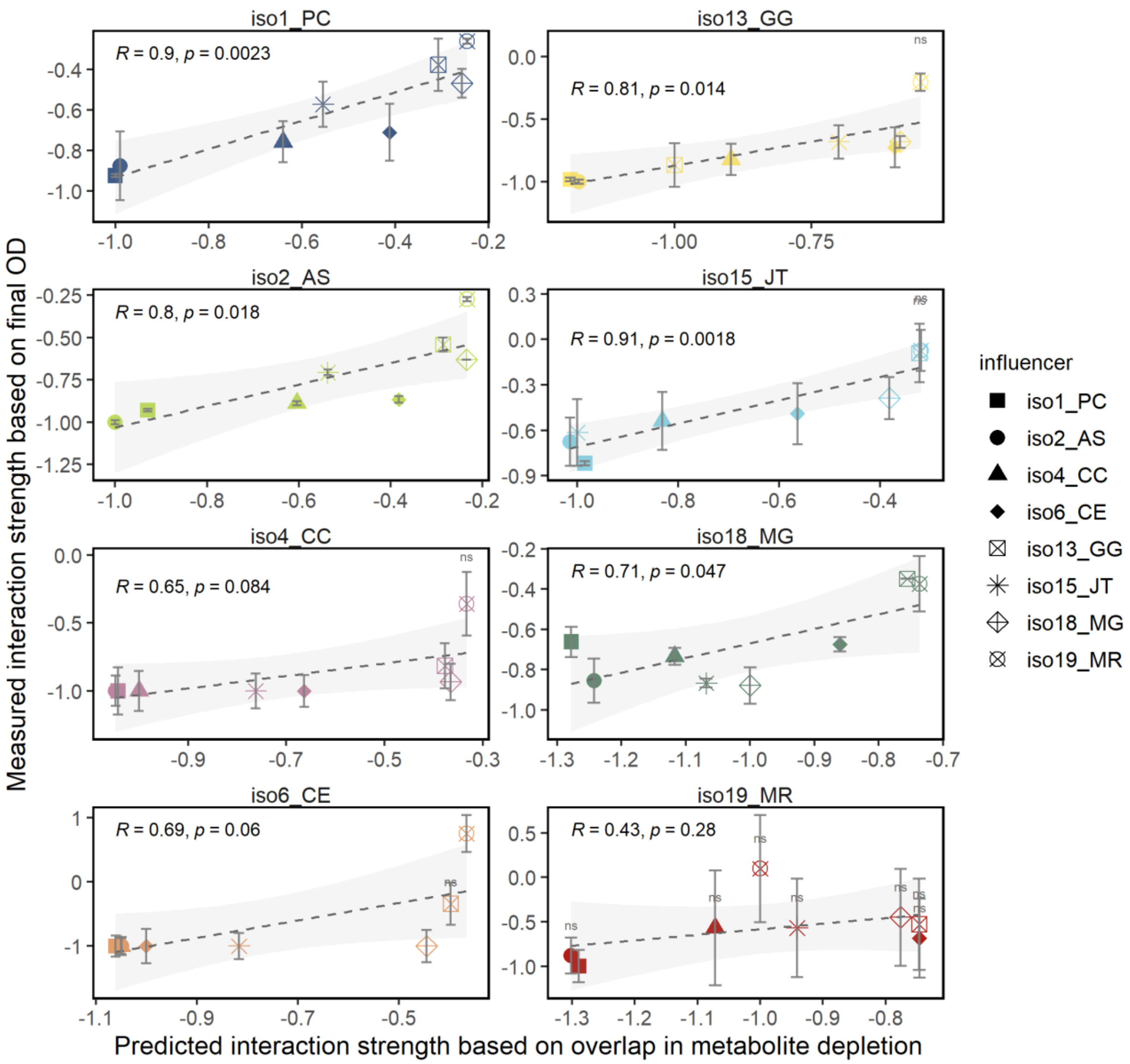

Supplementary Figure 6. Correlations between predicted and measured interaction strengths based on final OD. Each subplot represents results of different influencer isolates' effects on one recipient isolate. Predictions are calculated as the overlap in metabolite depletion between the recipient and influencer isolate (Figure 3E). Measured interaction strengths refer to the mean relative change in the recipient isolate's final OD when grown in the influencer isolate's spent medium compared with soil defined medium. Error bars represent standard deviations (n = 3). Points labeled "ns" meaning interaction not significant, that is recipient's final OD when grown in that influencer's spent medium did not change significantly as compared with in soil defined medium ($p > 0.05$, one-way ANOVA with post-hoc Dunnett's Test). Linear correlation R and p values are shown in each subplot.

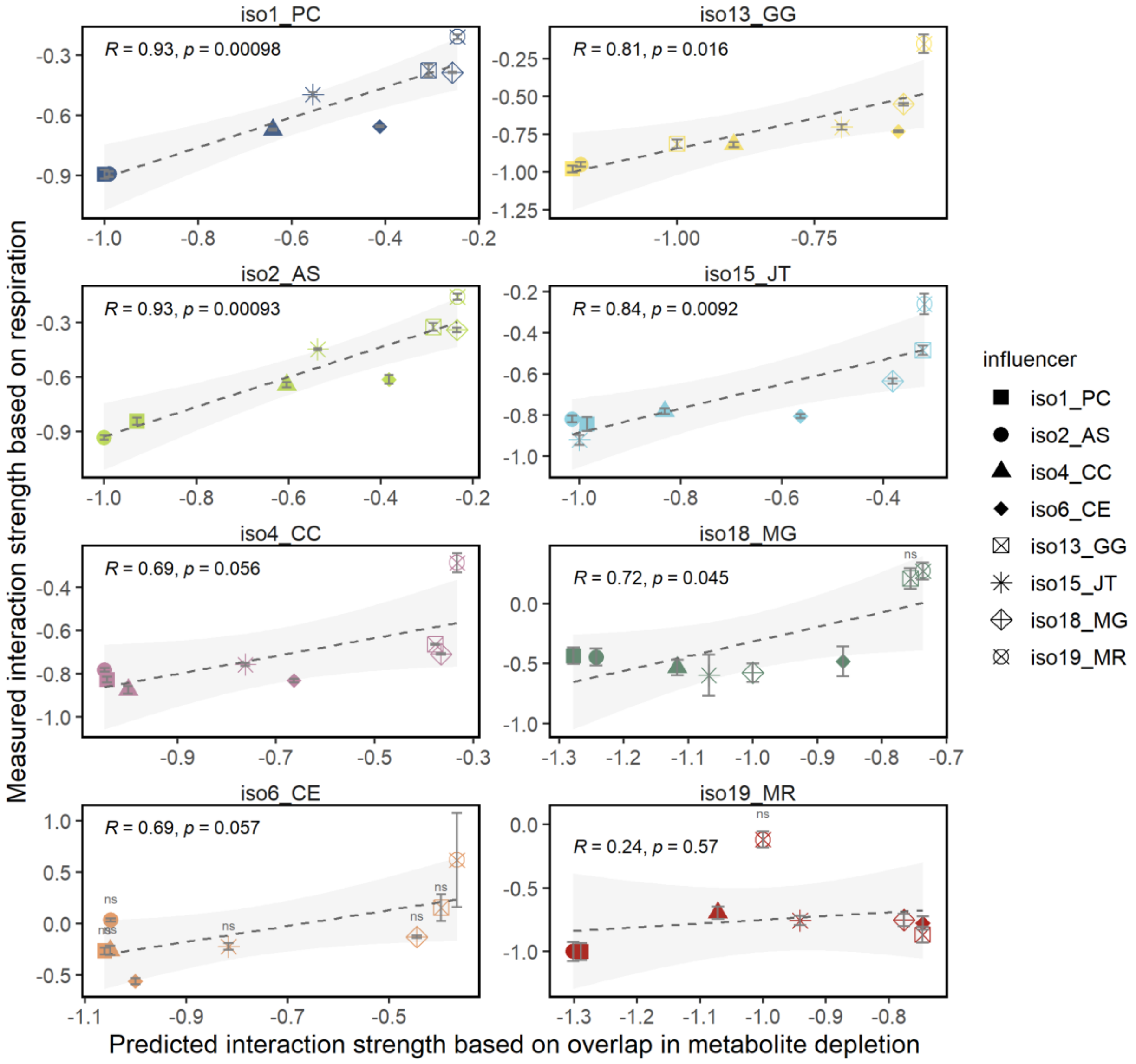

Supplementary Figure 7. Correlations between predicted and measured interaction strengths based on respiration. Each subplot represents results of different influencer isolates' effects on one recipient isolate. Predictions are calculated as the overlap in metabolite depletion between the recipient and influencer isolate (Figure 3E). Measured interaction strengths refer to the mean relative change in the recipient isolate's cumulative respiration when grown in the influencer isolate's spent medium compared with soil defined medium. Error bars represent standard deviations (n = 3). Points labeled "ns" meaning interaction not significant, that is recipient's respiration when grown in that influencer's spent medium did not change significantly as compared with in soil defined medium ($p > 0.05$, one-way ANOVA with post-hoc Dunnett's Test). Linear correlation R and p values are shown in each subplot.

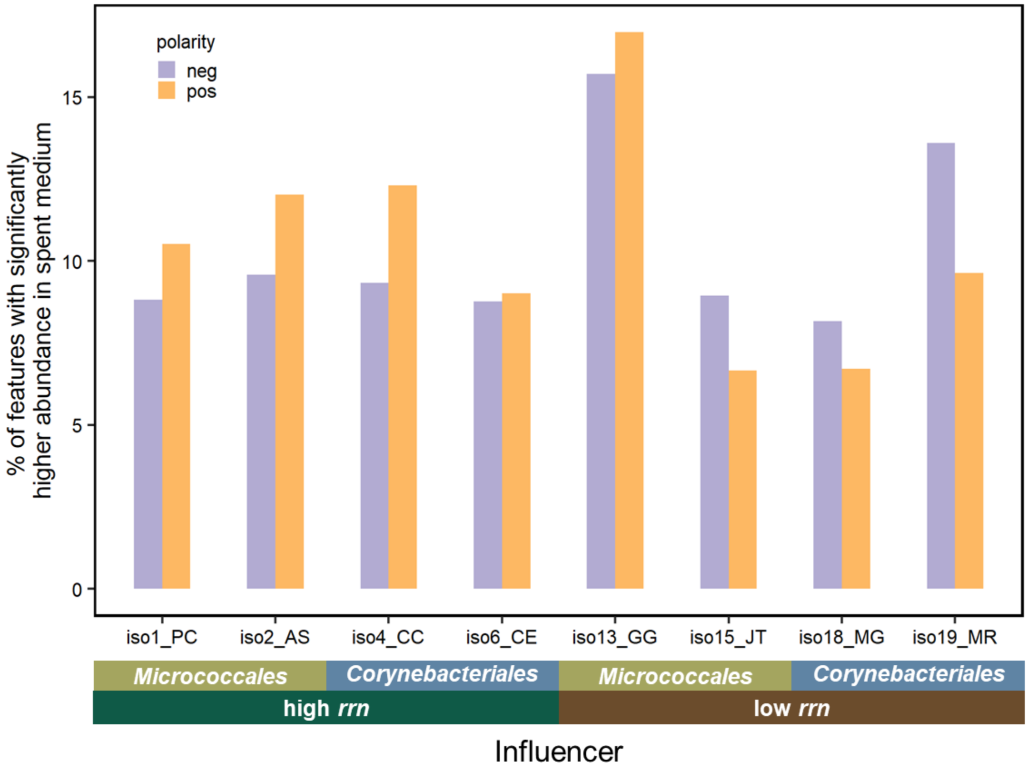

Supplementary Figure 8. Percentage of LC-MS features with significantly higher abundance in isolate spent medium than in soil defined medium control as determined using one-way ANOVA with post-hoc Dunnett's Test (n = 3, $p < 0.05$) under each positive and negative ionization mode. There were no significant effects of taxonomic or *rrn* copy group, or their interaction on the percentage of increased features by each isolate, under both polarities as well as the average result ($p > 0.05$, ANCOVA). Feature count results can be found in Supplementary Table 5.

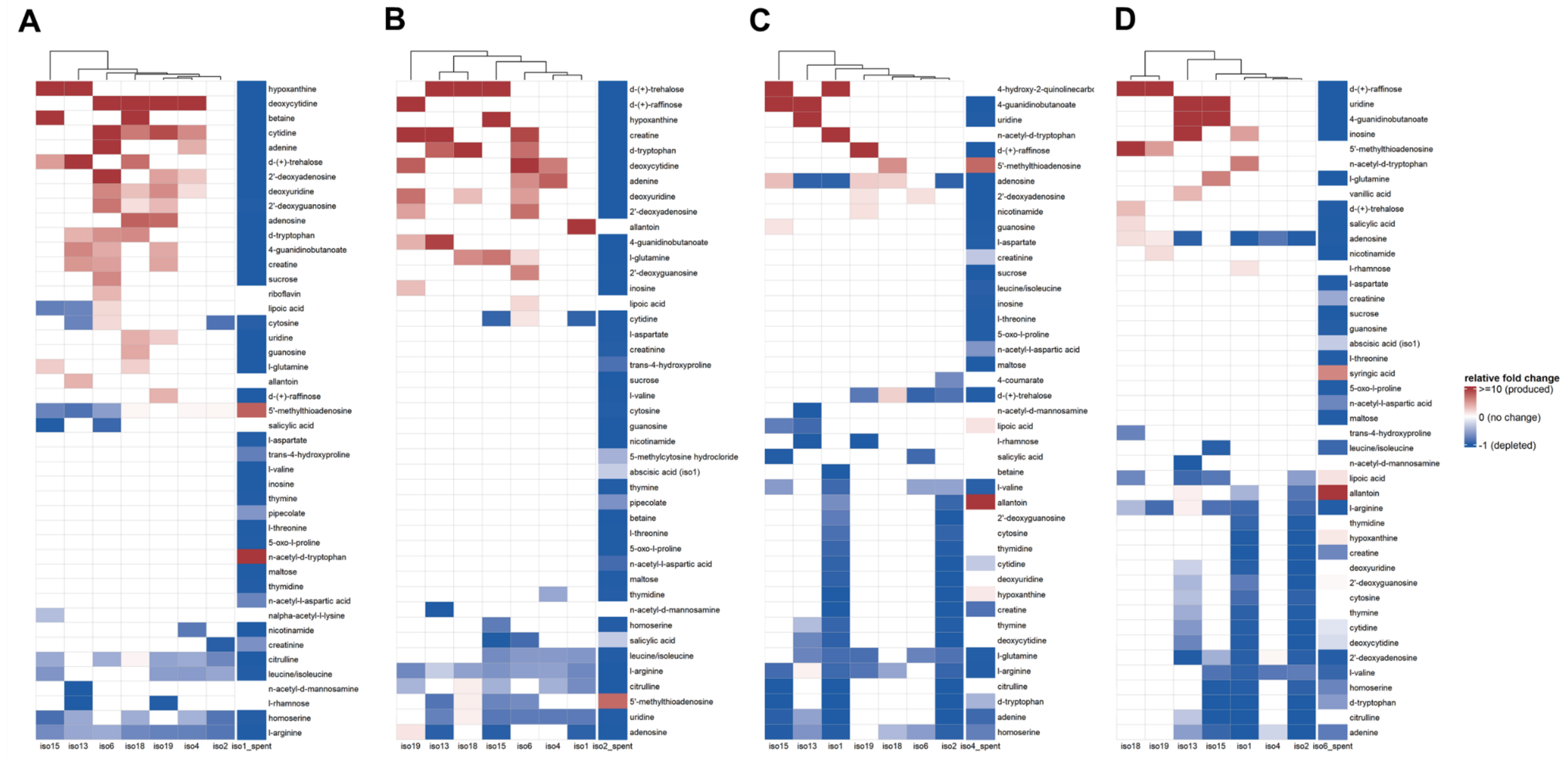

Supplementary Figure 9. Relative fold changes of metabolites in spent media of **(A)** iso1-*Pseudarthrobacter chlorophenolicus*, **(B)** iso2-*Arthrobacter sp.*, **(C)** iso4-*Corynebacterium callunae*, and **(D)** iso6-*Corynebacterium efficiens* before and after sequential growth experiments. In each heatmap, for example in **(A)**: the most right column "iso1_spent" represents metabolite changes in iso1's spent medium than in the soil defined medium; then iso1's spent medium was used to grow a second isolate and the resulting supernatant collected (double spent medium); metabolite changes in each double spent medium compared with the initial iso1's spent medium are displayed in the left seven columns. A positive (red) or negative (blue) value indicates production or depletion of that metabolite compared with the corresponding medium control, respectively. For compound whose abundance did not differ significantly, relative fold change was 0 and shown in white (n = 3, $p > 0.05$, one-way ANOVA with post-hoc Dunnett's Test). Only features annotated with high identification confidence (with matching *m/z*, retention time, and MS/MS spectra) are shown here.

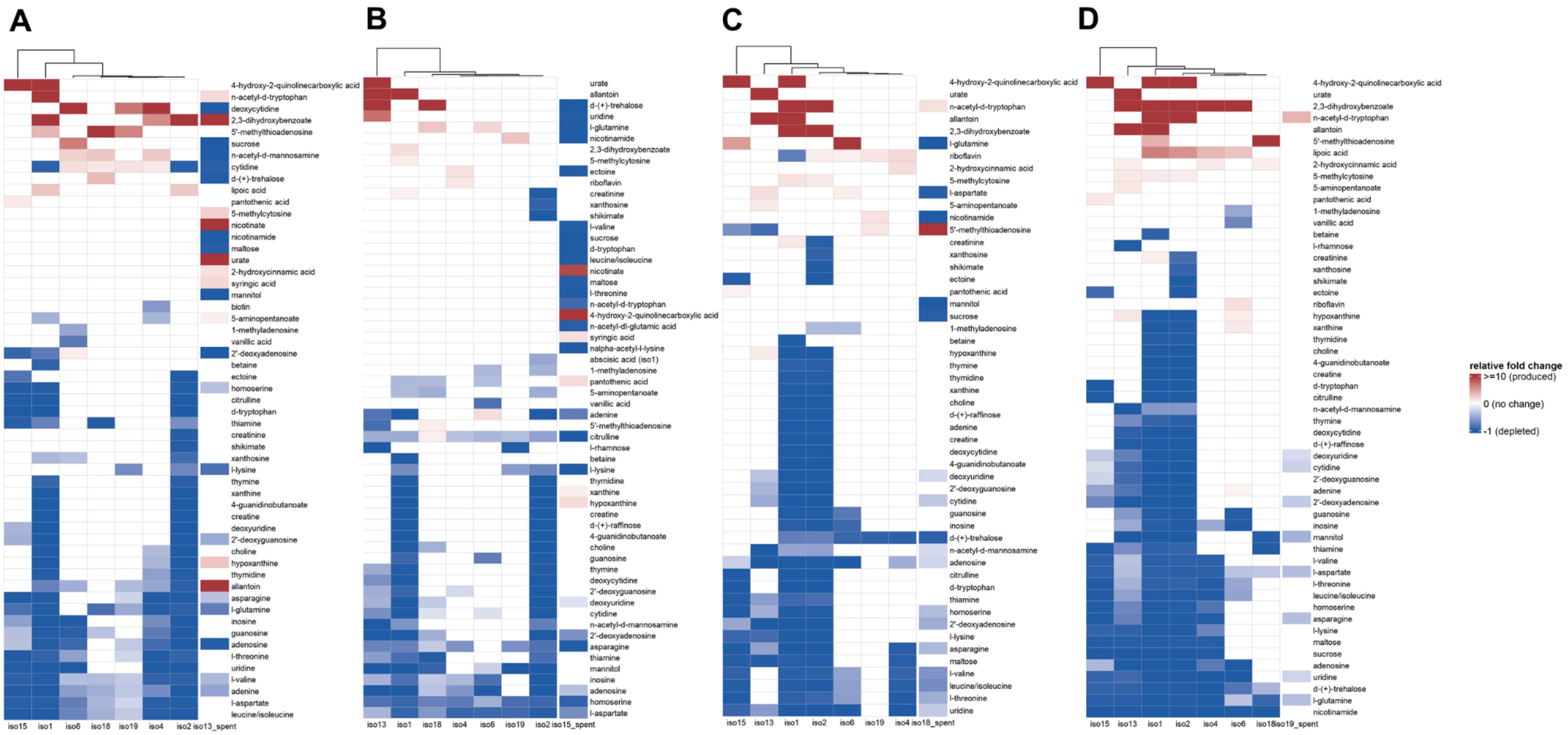

Supplementary Figure 10. Relative fold changes of metabolites in spent media of **(A)** iso13-*Gryllotalpicola ginsengisoli*, **(B)** iso15-*Janibacter terrae*, **(C)** iso18-*Mycolicibacterium gilvum*, and **(D)** iso19-*Mycolicibacterium rutilum* before and after sequential growth experiments. Similarly to Supplementary Figure 9, a positive (red) or negative (blue) value indicates production or depletion of that metabolite compared with the corresponding medium control, respectively. For compound whose abundance did not differ significantly, relative fold change was 0 and shown in white (n = 3, $p > 0.05$, one-way ANOVA with post-hoc Dunnett's Test). Only features annotated with high identification confidence (with matching *m/z*, retention time, and MS/MS spectra) are shown here.
